# Extending Injury- and Disease-Resistant CNS Phenotypes by Repetitive Epigenetic Conditioning

**DOI:** 10.3389/fneur.2015.00042

**Published:** 2015-03-02

**Authors:** Jeffrey M. Gidday

**Affiliations:** ^1^Department of Neurosurgery, Washington University School of Medicine, St. Louis, MO, USA; ^2^Department of Ophthalmology and Visual Sciences, Washington University School of Medicine, St. Louis, MO, USA; ^3^Department of Cell Biology and Physiology, Washington University School of Medicine, St. Louis, MO, USA

**Keywords:** stroke, neuroprotection, preconditioning, postconditioning, epigenetics, retina

## Abstract

Significant reductions in the extent of acute injury in the CNS can be achieved by exposure to different preconditioning stimuli, but the duration of the induced protective phenotype is typically short-lasting, and thus is deemed as limiting its clinical applicability. Extending the period over which such adaptive epigenetic changes persist – in effect, expanding conditioning’s “therapeutic window” – would significantly broaden the potential applications of such a treatment approach in patients. The frequency of the conditioning stimulus may hold the key. While transient (1–3 days) protection against CNS ischemic injury is well established preclinically following a single preconditioning stimulus, repetitively presenting preconditioning stimuli extends the duration of ischemic tolerance by many weeks. Moreover, repetitive intermittent postconditioning enhances post-ischemic recovery metrics and improves long-term survival. Intermittent conditioning is also efficacious for preventing or delaying injury in preclinical models of chronic neurodegenerative disease, and for promoting long-lasting functional improvements in a number of other pathologies as well. Although the detailed mechanisms underlying these protracted kinds of neuroplasticity remain largely unstudied, accumulating empirical evidence supports the contention that all of these adaptive phenotypes are epigenetically mediated. Going forward, additional preclinical demonstrations of the ability to induce sustained beneficial phenotypes that reduce the burden of acute and chronic neurodegeneration, and experimental interrogations of the regulatory constructs responsible for these epigenetic responses, will accelerate the identification of not only efficacious but also practical, adaptive epigenetics-based treatments for individuals with neurological disease.

## State of the Art

It is now widely established that organisms, tissues, and cells respond to sublethal stressors by a transient augmentation in their innate capacity to resist injury. That low doses of a harmful stimulus can promote beneficial responses forms the basis of hormesis theory (1), but only relatively recently have the conceptual and mechanistic connections between conditioning-induced tolerance and the hormetic response been recognized (2–5). In any event, seeking to leverage conditioning- or hormesis-based adaptive responses in patients suffering from acute ischemia, we remain somewhat stymied by several features of our laboratory models that seem to preclude easy translation to the clinic.

Significant progress has been made in preclinical models that advance our understanding of the molecular and genetic mechanisms underlying ischemic tolerance in the CNS (6–9). Many unique subcategories of this general phenomenon have been identified and subject to experimental scrutiny (8). For example, we recognize two basic phases or therapeutic windows for conditioning-induced protection: an early “rapid preconditioning” phase that is manifested nearly immediately after the adaptive stimulus is presented, which is largely dependent on rapid post-translational protein modifications, and a delayed preconditioning phase that takes several hours, sometimes days, to fully develop, resulting from changes in gene expression. We know that improved outcomes can result if the conditioning stimulus is applied to another tissue (“remote conditioning”), although its neural/humoral mechanism remains elusive. We have also learned that, even when initiated after the injurious event (“postconditioning”), conditioning stimuli can still afford protection. However, in all of these conditioning paradigms, we have defaulted to the assumption that the duration of the resultant injury-tolerant state requires a defined period of time to become established, and, more importantly, that it cannot be sustained indefinitely. This latter supposition has hindered, directly or indirectly, the clinical applicability of this phenomenon, as physicians face the daunting challenge of how best to pre- or post-condition patients at a well-defined higher risk for an ischemic event, let alone post-condition those stroke patients that present in the absence of recognizable risk factors. Leveraging such innate adaptive responses to treat chronic neurodegenerative disease seems even more unlikely, if not impossible.

Against this backdrop, I explore herein the contention that, as a result of epigenetics-based changes in gene expression, the duration of injury resistance following a stress stimulus can be increased quite significantly based on specific manipulations of the “dose” of the conditioning stimulus. Further interrogation of how best to establish protracted therapeutic windows of innate neuroprotection will provide some of the most valuable opportunities for multiplying the clinical potential of conditioning to prevent and treat both acute and chronic neurological disease.

## Redefining Stimulus-Response

Throughout most of its history at both the bench and in the clinic, the conditioning/tolerance field has evolved based on a fundamental, implicit definition of the very phenomenon we have come to understand: A transient, but robust, reduction in injury from acute ischemia can be realized if the tissue is preconditioned “X” hours prior to the lethal ischemic insult. For the aforementioned delayed phase of conditioning, the words “transient” and “X” in this definition have been understood to be ~8–48 h, essentially bookending the “therapeutic window” for treatment. Similarly, for postconditioning, preclinical protocols typically involve conditioning shortly after the ischemic event, as well as performing relatively early assessments of efficacy based largely on lesion quantification. Few studies have examined whether the duration of tolerance resulting from pre- or post-conditioning can be extended secondary to manipulating the “dose” of the conditioning treatment, or the related possibility that repetitive postconditioning during recovery can not only reduce injury but concomitantly enhance one or more long-term stroke recovery metrics; both of these advances would portend powerful clinical implications. In dose–response parlance, if the goal is to invoke a protracted response or prolonged period of tolerance to a given dose of conditioning, and if the given period of tolerance currently appears time-limited, then a closer examination of the conditioning dose is required to achieve this desired response. This has not really happened in any systematic sense; rather, we were quick to adopt the general assumption that the only type of tolerance is one defined by a short therapeutic window.

Every stimulus can be defined by at least three parameters: magnitude, duration, and frequency. Until the advent of postconditioning by instituting cycles of brief ischemia during early reperfusion, the vast majority of preconditioning paradigms for stroke have historically involved a single stimulus (i.e., frequency = 1) of a specific duration and magnitude that set into motion tolerance-signaling cascades; in the field of myocardial ischemia, repetitive preconditioning treatments have been explored (10), but not for the purpose of identifying a treatment that extends the duration of tolerance. Often overlooked is the fact that, like every stimulus, every response can also be defined by at least two of the same three parameters: its robustness and its duration. However, the preconditioning field settled quickly on a magnitude-focused, binary definition of tolerance as protection/no protection measured within ~24–72 h of conditioning; little attention has been focused on whether the duration of a given protective response – again, what is essentially the therapeutic window for this treatment – could be extended. The most obvious way to do so involves changes in stimulus frequency.

In fact, stimulus frequency is critical not only for determining the duration of the phenotypic change induced but also for its cumulative ability to promote an adaptive response, in contrast to the potential impotence of a single exposure or the potential injury resulting from multiple exposures combined as one or administered too closely together. Indeed, evidence emerging not only in the field of conditioning but across other dimensions of biology as well indicates that the frequency of the presented stimulus may be the key dose feature with respect to generating a beneficial, adaptive response instead of a harmful, maladaptive response (or no response at all). Exercise represents one example of benefit-inducing repetitive conditioning (11); moreover, recent clinical studies support the contention that increasing the number of short duration/low intensity bouts of exercise may provide more “advantages” to the cell, tissue, or organism than fewer exposures that are longer and/or more severe (and thus may afford no adaptive benefit or even cause injury) (12). As another example, repetitive intermittent hypoxia, initiated after incomplete spinal cord injury, promotes persistent functional recovery of respiratory and non-respiratory motor systems, as manifested by protracted improvements in gait performance in both rodents (13) and humans (14). In the field of psychology, the enhanced neuronal plasticity that promotes resilience – instead of vulnerability – to a number of different stress paradigms is becoming recognized as uniquely dependent on the frequency of exposure to the stressor (15–21). The long-term effects of intermittent fasting, and meal frequency and timing, on health and disease outcomes (22) represent additional facets of this same concept. Finally, repeated exposures to stress are also central to encoding a largely undefined but still well-recognized “resiliency” in medical, military, and law enforcement personnel and other individuals needing to perform well in adverse environments.

## Extending the Duration of Adaptive Change

With respect to stimulus frequency influencing the duration of the resultant phenotype, examples can be found in the conditioning literature that either strongly hint at such a possibility or support it directly. For example, the therapeutic window for myocardial ischemic tolerance resulting from a single hypoxic challenge was extended significantly if intermittent mild hypoxia is used as the conditioning stimulus (23, 24). Similarly, long-term thalamic atrophy following transient focal stroke in mice was attenuated by an intermittent hypoxic postconditioning stimulus initiated 5 days after ictus (25). In neonatal (26) and adult rats (27), multi-day hypoxic postconditioning reduced cerebral injury when the first challenge was initiated an hour after the severe hypoxic-ischemic insult. We found that, relative to the <3-days duration of ischemic tolerance induced in the mouse neural retina by a single hypoxic preconditioning stimulus, tolerance lasting at least 4 weeks after the last hypoxic preconditioning treatment can be realized if the hypoxic preconditioning challenge is repeated six times over a 2-week period (28). A similar extension of the therapeutic window (>2 months) for protection against murine cerebral ischemia is also afforded by a 2-week intermittent hypoxia preconditioning regimen (29), but only with stochastic increases in the frequency, duration, and intensity of the hypoxic stimulus relative to that which was efficacious in protecting against retinal ischemia. This distinction, and the examples above, not only suggest that tissue-, cell, age-, and species-dependent hormetic dose–response relationships may be operative but also suggest that stimulus frequency is not a simple concept in and of itself, and variations in the magnitude and duration of a given intermittently presented stimulus, as well as its temporal pattern, may make the difference between efficacy, impotence, and harm. The mixed preclinical track-record reported to date, wherein some repetitive conditioning protocols promote stroke tolerance, but not necessarily long-lasting stroke tolerance (30, 31), may be a reflection of this complexity. While the concept that extended periods of tolerance resulting from repetitive conditioning can be modeled in neuronal (32) and cerebral endothelial cell (33) cultures, the ultimate identification of a safe and efficacious, intermittent stimulus-based conditioning protocol – either physiologic or pharmacologic – that provides consistent, long-lasting protection in patients will prove extremely challenging in a diverse cross-section of human patients without reliable biomarker metrics to guide dosing regimens. Clearly, at this juncture, continued work in animal models is warranted.

Despite the above caveats, because most heart attacks and strokes are unpredictable, the inherent clinical advantage of postconditioning relative to preconditioning still generates considerable translational excitement. Progress might be accelerated if postconditioning protocols move beyond cyclical interruptions of early reperfusion to assessments of the benefits accruing from more protracted, repetitive treatments, and if long-term survival rates and functional metrics of post-stroke recovery become the more standard endpoints. Indeed, in cardiac ischemia, repeated remote postconditioning improved long-term survival relative to both a single preconditioning challenge and to an even lower frequency of repetitive remote postconditioning (34). Intentionally delaying the start of the repetitive postconditioning treatment volley may be prudent, given that, when initiated a week after stroke, moderate intermittent hypoxia rescued ischemia-induced impairments in learning and memory (35), whereas initiating the same treatment 1–2 days post-stroke increased mortality (36). Similarly, the aforementioned study of intermittent hypoxic postconditioning preventing post-ischemic thalamic atrophy was efficacious when the stimulus volley was initiated 5 days, but not 1 day, after ictus (25). Predictably, the optimal treatment window will likely vary depending on the age, species, injury type, and the postconditioning stimulus employed [including physical rehabilitation and exercise (37, 38)]. No matter what the tissue, rigorous, systematic investigations of multiple postconditioning “dose” regimens/protocols (34, 36, 39) – essentially time-consuming, iterative, titration-directed experiments – will be needed to identify both the dose and the optimal time after the acute injury to initiate treatment if this therapeutic approach is ever to be clinically implemented (40, 41). At present, we really know very little about the frequency of a given postconditioning stimulus needed to activate innate plasticity mechanisms capable of promoting and sustaining a neurorecovery-enhancing phenotype. We know less still about “preconditioning” treatments that effectively “post-condition” the stroked brain against a second stroke; repetitive stimuli may hold promise as the ideal treatment approach for this challenge. That said, some clinical trials of repetitive conditioning have moved forward empirically, and some have shown efficacy. As examples, hyperbaric oxygen treatments (5 days/week for 8 weeks) led to significant neurological improvements in both stroke (42) and head trauma (43) patients, even when the treatment was initiated many months after injury. And intermittent remote postconditioning by upper limb cuff inflation twice a day for 300 consecutive days proved effective in reducing the incidence of recurrent stroke in patients with symptomatic atherosclerotic intracranial arterial stenosis (44).

## Conditioning for Chronic Neurologic Disease

That distinct patterns of “continuous” but intermittent conditioning treatments provide protracted periods of protection against stroke and other acute neurological disorders implies that the slow and progressive neuronal death that defines neurodegenerative disease might also be significantly impacted by a similar therapeutic strategy. Evidence from the hormesis field that repeated presentations of low-dose stressors extend beneficial, prevention-, and/or recovery-promoting effects in preclinical models of more chronic cardiovascular, immunological, metastatic, and other non-neurological and non-psychological pathologies, as well as in human studies (4), supports this contention. Not surprisingly then, when tested, the intermittent hypoxia protocol that we leveraged in mice to extend the duration of tolerance to acute retinal ischemia (28) also proved efficacious as a preconditioning stimulus against glaucoma, significantly reducing retinal ganglion cell somal and axonal loss at 3 and 10 weeks of disease in mice (45). Moreover, significant improvements in these same cell survival metrics were realized when we postconditioned mice with a repetitive intermittent hypoxia protocol initiated after disease onset (46). Repetitive postconditioning with brief bouts of retinal ischemia was also efficacious in reducing retinal neurodegeneration in rat models of glaucoma (47) and diabetic retinopathy (48). Suggestive of potential efficacy in Alzheimer’s, intermittent hypoxic postconditioning reduced oxidative and nitrosative stress metrics and improved morphologic and functional (memory) outcomes following intracerebral injections of amyloid beta (49). Although not widely realized, an extensive number of preclinical and clinical studies in the Soviet Union, in which an estimated two million individuals were treated over long periods of time with intermittent hypoxia, document a variety of health-promoting and injury-resistant outcomes from such treatments, in the absence of adverse effects (50). Collectively, these findings provide strong support for the hypothesis that intermittent conditioning during the progression of chronic neurological disease may provide a means of both inducing and “holding on” to an adaptive phenotype that in turn is manifested not only as a reduction in the kinetics of disease progression but also as improvements in functional status and long-term survival.

## Lifespan Effects

As alluded to above, the idea that repetitive stress conditioning can trigger and possibly maintain long-lasting phenotypic change in the CNS actually derives strong historical support from the neuropsychology and behavior fields, which provide rich examples of sustained plasticity within and across age groups, and ideal opportunities for considering stress along a negative–positive continuum, with distress anchoring one end and “eustress” anchoring the other (51). For instance, when initiated during adulthood, repeated mild stress exposures lead to persistent decreases in behavioral disorders and improvements in memory (52, 53). And the literature is actually replete with studies documenting adaptive phenotypes persisting into adulthood as a result of intermittent, mild stress challenges only experienced by the individual during intra-uterine or post-uterine development. This concept was introduced over 60 years ago based on the serendipitous findings of Levine (54), and is somewhat akin to the immunological concept of early childhood vaccinations for conferring life-long protection against disease. As it turns out, most research over the ensuing decades has focused on documenting illness phenotypes secondary to child or adolescent stress in an attempt to understand the origins of psychiatric disease (55). Nevertheless, reflecting yet another manifestation of “the dose makes the poison” and new ways of thinking about evolutionary fitness, there are still some investigations to be found of beneficial adult phenotypes resulting from exposing the same individual, when much younger, to a given stressor and/or exposing their caregiving mothers to stress (56, 57). Hypoxia-related examples of the former include the finding that postnatal mice exposed from birth to 4 weeks of age to mild intermittent hypoxia exhibit improved spatial learning and memory during their adult life (58), and the report that brief hypoxic challenges during neonate life confer resistance to senescence and better preservation of cognitive function in aged rats (59). Resilience to stress in adult mice raised by mothers with access to postpartum exercise (60) is an example of the latter. Intermittent separations of baby monkeys from their mother may represent a form of “stress inoculation” that leads to enhanced arousal regulation and resilience of these offspring in later life (15). There is also evidence that unpredictable, stochastic stress exhibits unique age-dependent effects, promoting future resilience when experienced by juvenile animals, but not by adults (61). Given these and other provocative findings, some have even proposed – for humans – intentional exposures to intermittent eustress (like mild hypoxia) during neonatal or adolescent periods as a way to prevent or lessen future disease burden (62).

## Epigenetics as Primary Mechanism

The molecular basis of the many adaptive responses to repeated stress highlighted thus far is predictably complex, given the relative permanence of the resultant phenotype. Many studies have sought to characterize the beneficial phenotypes, finding evidence for increased neurogenesis (53), changes in hormonal balance (15, 18), changes in modulators of synaptic plasticity (63, 64), changes in sodium–calcium exchangers (NCX) (65), and elevated levels of HIF gene target mRNAs/proteins (52) or other survival factors (31), to name a few. While identifying these phenotypes is interesting with respect to understanding how disease resistance is ultimately manifested, it is epigenetics that deserves attention as the fundamental mechanism responsible for the long-lasting responses to repetitive conditioning stimuli, and thus the likely target of future therapeutics. In brief, epigenetics involves the regulatory processes – DNA methylation and changes in chromatin structure secondary to post-translational histone modifications – that reside “above” the level of genes and control their readout. During development, epigenetics specifies cell fate determination and perpetuation, but we now know that these same mechanisms are engaged throughout the lifespan by “experience” or “environment,” in all their different forms. Importantly, considerable evidence indicates that the changes in gene expression resulting from these stable covalent DNA modifications or epigenetic “marks” can be long-lasting, and, in some instances, endure throughout the lifetime of post-mitotic cells; some may even persist through cell division and be transmitted via the germline to future generations (see below). Thus, epigenetics is really the biochemically driven interface between nature (genes) and nurture (all manner of environmental and behavioral/psychological stresses or exposures). Proximal to this interface, so to speak, distinct features of an experience or a stressor (e.g., frequency, severity, etc.) that, as alluded to earlier we have only recently begun to dissect and define with respect to threshold and interactive effects, dynamically modify the epigenome. Moreover, epigenetic marks can accumulate over the course of multiple exposures and then act collectively to determine a new homeostatic phenotypic set-point. Distally, after encoding molecular memories of these experiences and exposures, CNS function and behavior is altered secondary to changes in gene expression, thus impacting one’s vulnerability or resilience to future stressors/disease (66). In essence, epigenetics is really the “hard-wired” evolutionary response for successful adaptation to changing natural and social environments. With the realization that phenotype is fluid, and not rigidly defined by genotype, it is not an exaggeration to claim that broadening our understanding of “neuroepigenetics” (67) and its regulation at both cellular and organismal levels holds incredible promise for reducing CNS disease burden and enhancing neurological health.

The ischemia-tolerant phenotype resulting from a pre- or post-conditioning stimulus is, by definition, an epigenetic response, but only recently have we begun to directly recognize it as such (68–70). Genomic and proteomic analyses consistently reveal that the ischemia-tolerant CNS is defined by a broad transcriptional repression (6, 68, 71) – in effect, the manifestation of an epigenetically mediated response to the conditioning stimulus. Tests of the hypothesis that members of the evolutionarily conserved polycomb protein family – known repressors of gene transcription secondary to their ability to posttranslationally modify histones and thus maintain them in an inactive state (72) – may be causal epigenetic mediators ultimately responsible for establishing this metabolically downregulated phenotype confirmed that increases in polycomb group proteins define the “traditional” ischemia-tolerant CNS (68, 73). Certainly, other transcriptional regulators of gene repression – such as repressor element-1 silencing transcription factor (REST) (74) – may also be involved in orchestrating this response. Whether polycomb and/or REST proteins and/or others participate in mediating the longer periods of injury resistance induced by repetitive pre- or post-conditioning stress paradigms has yet to be examined. Additional studies uncovering other epigenetic features of conditioning-induced tolerance have been forthcoming (75).

A rapidly evolving subfield of epigenetics extends the aforementioned phenomenon of even life-long phenotypic change within an individual to the transmission of adaptive or maladaptive phenotypes from parents to their offspring. Epigenetic inheritance, or transgenerational epigenetics, provides a mechanistic foundation for Lamarck’s notion that the effects of environment and/or experience could be inherited by, and thus benefit, one’s immediate offspring – known as “soft inheritance” in Lamarck’s day. This phenomenon is not inconsistent with Darwinian evolutionary theory, given the selection pressure inherent in preserving adaptive phenotypic variation if it increases fitness when resources and/or other environmental stress levels change; but it does carry the important implication that random DNA mutations are not the sole driving force of evolution. In transgenerational epigenetics, gene expression is altered, not genetic inheritance; environment becomes heredity. At least in theory, this suggests the possibility of obtaining desired phenotypes in offspring that prevent or reduce disease burden and/or enhance vitality by therapeutically manipulating epigenetic regulatory systems in their parents. Conditioning across generations might seem at first blush to represent an implausible extension of tolerance’s therapeutic window. However, while the vast majority of preclinical studies have focused on the germline linking of adverse or dysfunctional behavioral changes in an individual to life experiences of their parents or grandparents (76–78), a small number of reports to date provide convincing examples, in mammals, of the transgenerational persistence of beneficial, epigenetically acquired phenotypes (79–82). Moreover, there is very intriguing epidemiological evidence that this occurs in humans (80, 83–85). Obviously, understanding the mechanistic basis of both potential outcomes portends huge repercussions for evolutionary biology, and will likely change the face of our understanding of the genetic basis of neurological disease.

## Conclusion

Despite the discovery of the robustness of endogenous cytoprotection almost 25 years ago, the thousands of published preclinical successes, and even the dozens of efficacious studies in humans, the time-limited therapeutic window that has inadvertently come to define conditioning-based responses continues to constrain its acceptance as a viable therapeutic strategy, even for acute tissue injury. However, given the accumulating evidence supporting long-lasting, epigenetics-mediated changes in phenotype in the CNS and other tissues secondary to repetitive stress, perhaps it is time to shelf the assumption that conditioning-induced tolerance is “transient,” reevaluate our assumptions about the defining features of tolerance, and in so doing open new doors regarding its full clinical destiny. As scientists and physicians, capturing the ability to leverage sustained, beneficial phenotypic changes capable of providing protracted periods of resilience to acute and chronic neurological injury should be our next collective goal.

## Conflict of Interest Statement

The author declares that the research was conducted in the absence of any commercial or financial relationships that could be construed as a potential conflict of interest.
